# Identification of longevity compounds with minimized probabilities of side effects

**DOI:** 10.1007/s10522-020-09887-7

**Published:** 2020-06-19

**Authors:** Georges E. Janssens, Riekelt H. Houtkooper

**Affiliations:** Laboratory Genetic Metabolic Diseases, Amsterdam UMC, University of Amsterdam, Amsterdam Gastroenterology and Metabolism, Amsterdam Cardiovascular Sciences, Meibergdreef 9, 1105 AZ Amsterdam, The Netherlands

**Keywords:** Geroprotectors, Spermidine, d-Glucosamine, Side effects, Hormesis

## Abstract

It is hypothesized that treating the general aging population with compounds that slow aging, geroprotectors, could provide many benefits to society, including a reduction of age-related diseases. It is intuitive that such compounds should cause minimal side effects, since they would be distributed to otherwise healthy individuals for extended periods of time. The question therefore emerges of how we should prioritize geroprotectors discovered in model organisms for clinical testing in humans. In other words, which compounds are least likely to cause harm, while still potentially providing benefit? To systematically answer this question we queried the DrugAge database—containing hundreds of known geroprotectors—and cross-referenced this with a recently published repository of compound side effect predictions. In total, 124 geroprotectors were associated to 800 unique side effects. Geroprotectors with high risks of side effects, some even with risk for death, included lamotrigine and minocycline, while compounds with low side effect risks included spermidine and d-glucosamine. Despite their popularity as top geroprotector candidates for humans, sirolimus and metformin harbored greater risks of side effects than many other candidate geroprotectors, sirolimus being the more severe of the two. Furthermore, we found that a correlation existed between maximum lifespan extension in worms and the likelihood of causing a side effect, suggesting that extreme lifespan extension in model organisms should not necessarily be the priority when screening for novel geroprotectors. We discuss the implications of our findings for prioritizing geroprotectors, suggesting spermidine and d-glucosamine for clinical trials in humans.

## Introduction

**Fig. 1:**
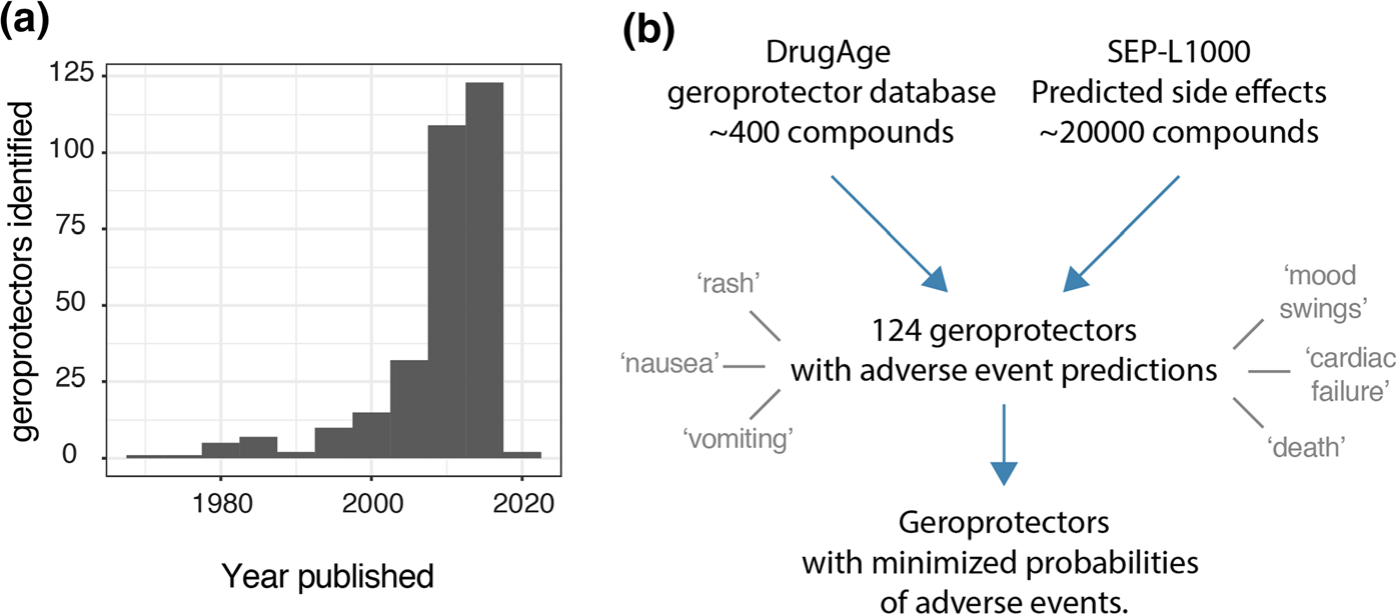
Analysis approach. **a** Geroprotectors listed in DrugAge with histogram of publication dates. **b** Analysis approach whereby compounds from DrugAge were cross-referenced for their predicted side effects based on the SEP-L1000 predictions database. 124 compounds overlapped in this way and were assessed for their predicted side effects, which ranged from terms such as ‘rash’ to ‘death’

Treating the general aging population with compounds that slow aging, geroprotectors, could provide many benefits to society. Specifically, it is believed that such compounds could reduce the onset of age-related diseases and thereby result in an extension of the healthy years of life. Meanwhile, the identification of novel compounds that extend lifespan in model organisms has been accelerating at an unprecedented rate, as can be visualized by the geroprotector entries from the DrugAge database (Barardo et al. 2017), plotting number of drugs registered per publication date (Fig. 1a). This phenomenon is likely due to a variety of factors, including the increased use of short lived model organisms such as *Caenorhabditis elegans* (worms), the invention and implementation of higher throughput technologies to assess lifespans, and computational drug screening approaches helping to identify novel geroprotectors (Stroustrup et al. 2013; Carretero et al. 2015; Janssens et al. 2019; Calvert et al. 2016; Petrascheck et al. 2007; Ye et al. 2014).

However, the ultimate aim of these efforts is to identify compounds that promote healthy aging in humans. The translatability of geroprotectors from model organism studies to humans requires minimally that the compounds are safe for use, without causing serious side effects or health issues. This is not always the case for geroprotectors, even when assessed in model organisms. For example, the compound lamotrigine extends lifespan in flies, though the same study also found compromised health in treated animals (Avanesian et al. 2010). Following such examples, the translatability of geroprotectors from model organisms to humans has been an ongoing topic of discussion, with a need for criteria to prioritize compounds. Recent work in the field of aging research has consolidated a primary set of criteria to help prioritize geroprotectors for human use, which states that these compounds should have been demonstrated to (i) increase lifespan, (ii) ameliorate human aging biomarkers, (iii) have acceptable toxicity, (iv) cause minimal side effects at therapeutic dosage, and (v) improve health-related quality of life (Moskalev et al. 2016).

Minimizing side effects can be considered one of the highest priorities to answer the question ‘what known longevity interventions should we test in humans.’ This is especially true considering that geroprotectors may be distributed widely throughout the population to otherwise healthy individuals, and possibly for an extended period of time. Furthermore, prioritization of compounds for clinical testing is essential since evaluation of even a single compound, such as is the case with metformin in the TAME study, may require estimations of roughly 75 million USD in funding (De Grey 2019; Barzilai et al. 2016). While various curated efforts have selected candidate compounds to test in humans—such as with the selection of metformin (Barzilai et al. 2016; Moskalev et al. 2016)—a systematic account addressing potential side effects of known geroprotectors to date has been lacking. This is largely due to the fact that most geroprotectors identified thus far have been identified in model organisms and are compounds considered ‘for research use.’ These compounds therefore simply do not possess a large base of human users who register their side effects. For example, of the > 400 compounds cataloged in the DrugAge database of geroprotectors, only ~ 11% are from studies performed in vertebrates, while the vast majority are from invertebrate models, *C. elegans* specifically accounting for several hundreds of the compounds that have been identified. Similarly, over 80% of DrugAge’s compounds are not considered as approved for human use when cross-referencing with the listing of drugs in DrugBank (Wishart et al. 2018). Furthermore, though geroprotectors may be repurposed from already FDA approved drugs and therefore possess safety profiles, cross-comparison of registered side effects with other geroprotectors for systematic evaluation has not been thoroughly performed.

Recently, databases have been published that allow overcoming these main obstacles. In addition to the DrugAge database cataloging longevity compounds, an account of the probabilities of side effects of ~ 20,000 small molecules has also been published (Wang et al. 2016), based on the L1000 database of transcriptional signatures (Subramanian et al. 2017). The SEP-L1000 as it is called (for “side effect prediction based on L1000 data”), derives probabilities of side effects of compounds based on their transcriptional profiles in human cell culture. In conjunction with this, the SEP-L1000 was built, amongst other resources, also based on the ‘Side Effect Resource’ (SIDER) database of reported side effects in humans (Kuhn et al. 2016). We hypothesized that this SEP-L1000 database (Wang et al. 2016), paired with the well-established DrugAge database cataloging known geroprotectors (Barardo et al. 2017), could allow us to assess the risks of side effects of gerorpotectors in a systematic manner.

## Methods

### List of lifespan extending drugs

DrugAge Build 2 (Barardo et al. 2017) (release date September 1, 2016, 1316 entries, 418 unique drugs, see also: https://genomics.senescence.info/drugs/index.php) was downloaded and lifespan extending drugs were defined as entries that had at least one average lifespan change entry that was greater than 0, and were listed as ‘yes’ in the ‘significance’ data column, which generally corresponded to a reported p value < 0.01. We note that in the case of spermidine published p values for lifespan extension in *M. musculus* (mice) were 0.018 and 0.012 and were listed in DrugAge as nonsignificant and therefore did not pass our inclusion criteria in our unbiased analysis (requiring a DrugAge listing of ‘significant’). Mouse effects for spermidine were therefore not included in Fig. 3d. Otherwise our findings remained unaffected. Manual curating led us to include mice in spermidine’s overview in Fig. 4b. To assess lifespan change induced by a geroprotector in DrugAge we used average lifespan rather than maximal lifespan since maximal lifespan is highly dependent on the number of organisms used in a lifespan study, with a greater n allowing for a higher chance of reaching a true maximum lifespan, and average lifespan is more robust against this. If compounds contained multiple entries in DrugAge, the entry that had the highest average lifespan was used in a species-specific manner.

### List of side effects for lifespan extending drugs

The compounds from DrugAge were manually checked for their presence in the SEP-L1000 database (Wang et al. 2016) (see https://maayanlab.net/SEP-L1000/). This was required due to the alternative naming of compounds sometimes used between databases. If any compound contained multiple entries in the SEP-L1000 database, the first entry was used. For each drug identified as having a side effect listing, the side effect predictions and their probabilities of occurrence were stored for further processing.

### Data processing and statistics

Total side effects per DrugAge compound were quantified by summing the number of SEP-L1000 side effects listed per compound. Maximum probability of a side effect for each DrugAge compound was quantified by selecting the highest SEP-L1000 side effect probability entry listed per compound. Association and significance between lifespan change and side effect values was tested for using Pearson’s product moment correlation coefficient. Data processing and visualizations were done using R (The R Development Core Team 2010) version 3.5.1 and the GGPlot2 package (Wickham 2011).

## Results

### Predicted side effects of geroprotectors

**Fig. 2:**
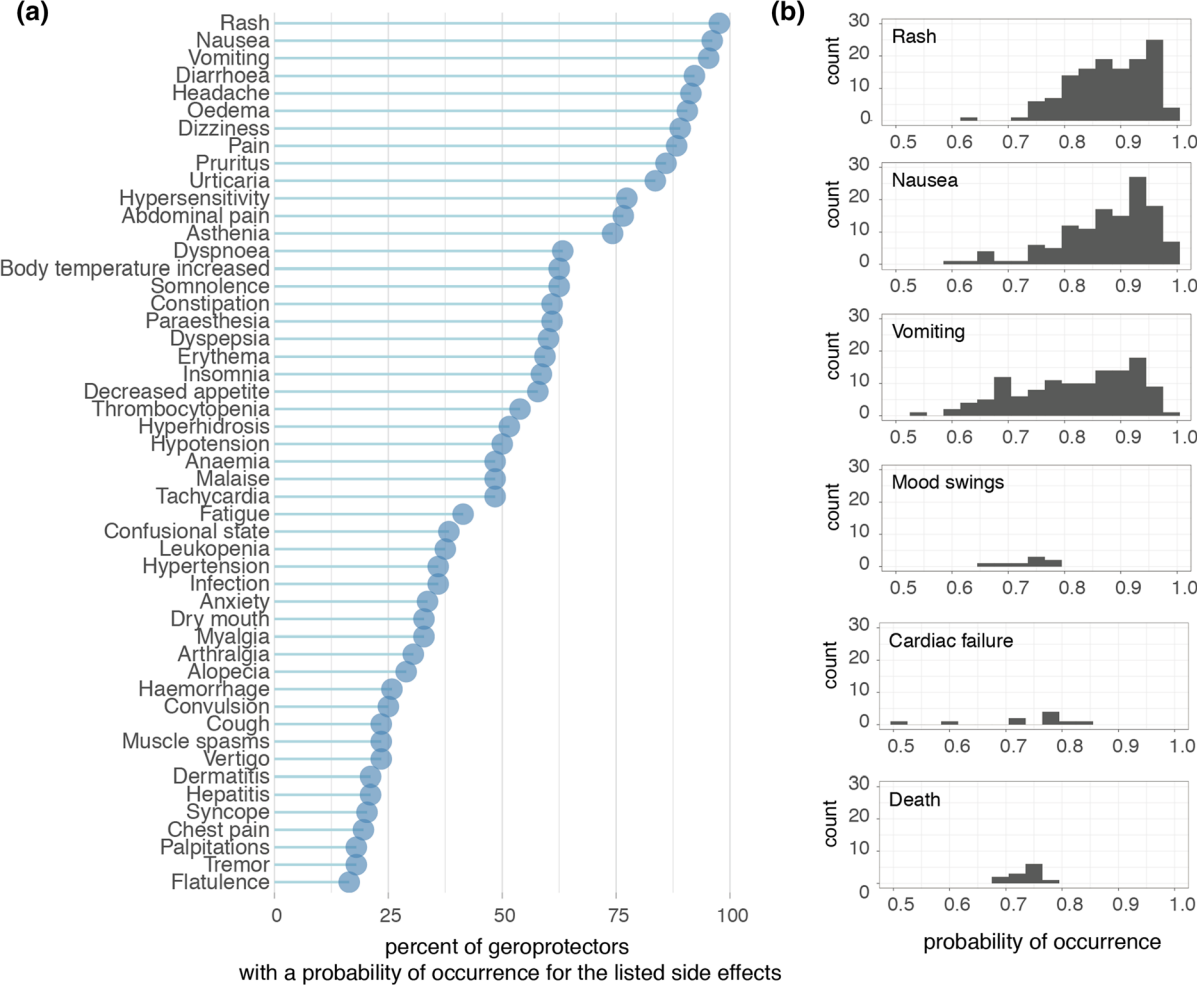
Side effects of geroprotector compounds. **a** Top 50 side effects as ranked by percent of appearance within the 124 drugs. Graph depicts the percent of geroprotectors with a probability of occurrence for the listed side effects. The probability of rash, nausea, and vomiting occurs in nearly all geroprotectors, though each geroprotector may have a different probability for the occurrence of these side effects. The distributions of these probabilities for selected side effects are depicted in (**b**). **b** Examples of the distributions of probabilities for six selected side effects; rash, nausea, vomiting, mood swings, cardiac failure, and death

At the time that we accessed the DrugAge database, over 400 unique compounds were listed as extending lifespan in at least one model organism. Subsequently cross-referencing this against the SEP-L1000 database which contained ~ 20,000 compound’s predicted side effects, we found an overlap of 124 compounds to which we could assign 800 unique side effects (Fig. 1b). In order to explore the nature and prevalence of these side effects amongst geroprotectors, we ranked side effects according to how often they were predicted to occur (Fig. 2a). We found that nearly all (> 95%) geroprotectors in our list had a > 50% probability of causing side effects such as rashes (SIDER ID C0015230), nausea (SIDER ID C0027497), or vomiting (SIDER ID C0042963) (Fig. 2a, b). While these may be termed ‘mild’ side effects, they nonetheless would greatly decrease quality of life in otherwise healthy individuals. Furthermore, several geroprotectors, albeit few, also included much more severe side effects of mood swings (SIDER ID C0085633), cardiac failure (SIDER ID C0018801), or even death (SIDER ID C1306577) (Fig. 2b). Clearly, these are undesirable on the long term, with the side effect of ‘death’ even being something directly opposite to the goal of geroprotection. This serves to highlight the importance of selecting geroprotectors with few total side effects or low probabilities of causing such effects.

Geroprotectors in general were not more prone to causing side effects compared to other compounds. When considering all 20,412 compounds in the SEP-L1000 database, similar probabilities of occurrence were observed for the most prominent side effects. Rash (associated with 20,198 compounds), nausea (associated with 20,166 compounds), and vomiting (associated with 19,937 compounds) were all predicted to occur for the vast majority of compounds (> 95%), while mood swings (associated with 149 compounds), cardiac failure (associated with 268 compounds), and death (associated with 198 compounds) were predicted for only a minority of the compounds (< 5%). Therefore, assessing side effects in geroprotectors serves rather to reveal and reinforce the fact that geroprotectors are not a drug class apart from others or free of side effects due to their longevity-inducing properties. Rather, careful and further consideration should be given to this class of compounds to ensure the lowest chance of side effects when used in humans.

**Fig. 3:**
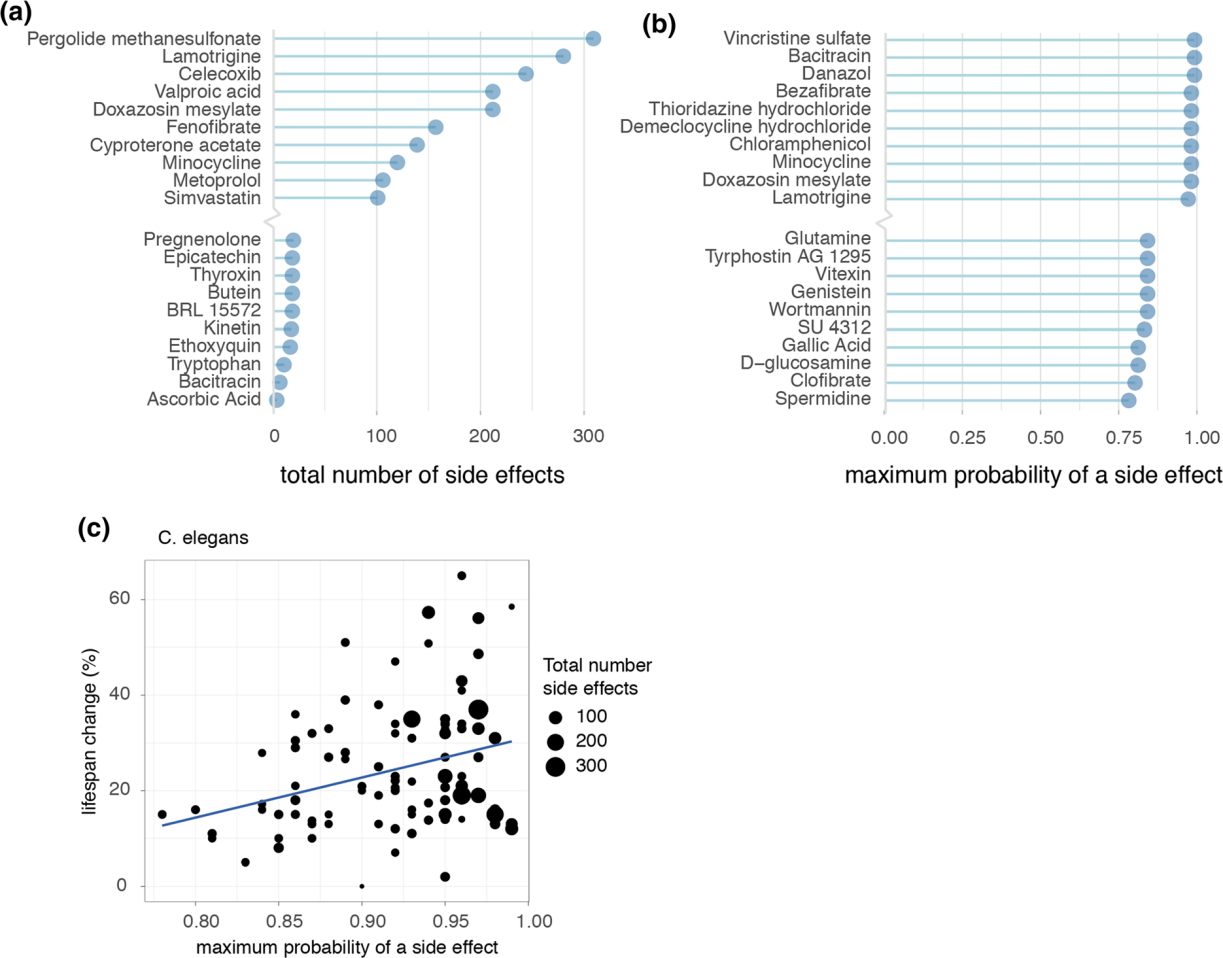
Longevity effects of geroprotectors relate to maximum likelihoods of causing side effects. **a** Top 10 highest and lowest ranked geroprotectors in relation to the total number of side effects they are predicted to produce. **b** Top 10 highest and lowest ranked geroprotectors in relation to the maximum probability of a side effect. **c** Positive correlation observed in worms (*C. elegans*) between geroprotector’s lifespan extension and probability of causing a side effect (R = 0.32, p = 0.0018)

In order to form a general overview of how dramatic an individual geroprotector’s risks of side effects were, we summarized for each geroprotector both the total number of side effects, and the highest probability that a side effect would be caused, according to the SEP-L1000 side effects database. Ranking compounds in these manners revealed those with both high and low risks for causing side effects (Fig. 3a, b). For example, lamotrigine, which was described to extend lifespan in flies though decrease health (Avanesian et al. 2010), had > 250 total predicted side effects listed, one of the highest in our ranking (Fig. 3a). Conversely, ascorbic acid (vitamin C), considered safe for humans even in high doses, was predicted to only cause three side effects (Fig. 3a). Likewise, lamotrigine had a nearly 100% maximum likelihood of causing a side effect, while spermidine, an autophagy inducer considered relatively safe for human use (Moskalev et al. 2016), carried only a 78% maximum likelihood of causing a side effect, the lowest in our analysis (Fig. 3b).

Assessing compounds in this way, we were able to inquire whether side effects in general possessed any correlation to the drug’s ability to increase lifespan. To address this, we turned to the *C. elegans* subset of DrugAge, since comparing geroprotector’s lifespan effects is more fairly performed within a single species, and *C. elegans* contained by far the largest repository of geroprotectors. Here, we plotted the maximum likelihood a compound had to cause a side effect relative to the percent it was able to increase lifespan. Remarkably, we found a significant positive correlation between a compound’s side effect risk and ability to increase lifespan (Fig. 3c) (R = 0.32, p < 0.01).

### Candidate geroprotectors for clinical trials with humans

**Fig. 4:**
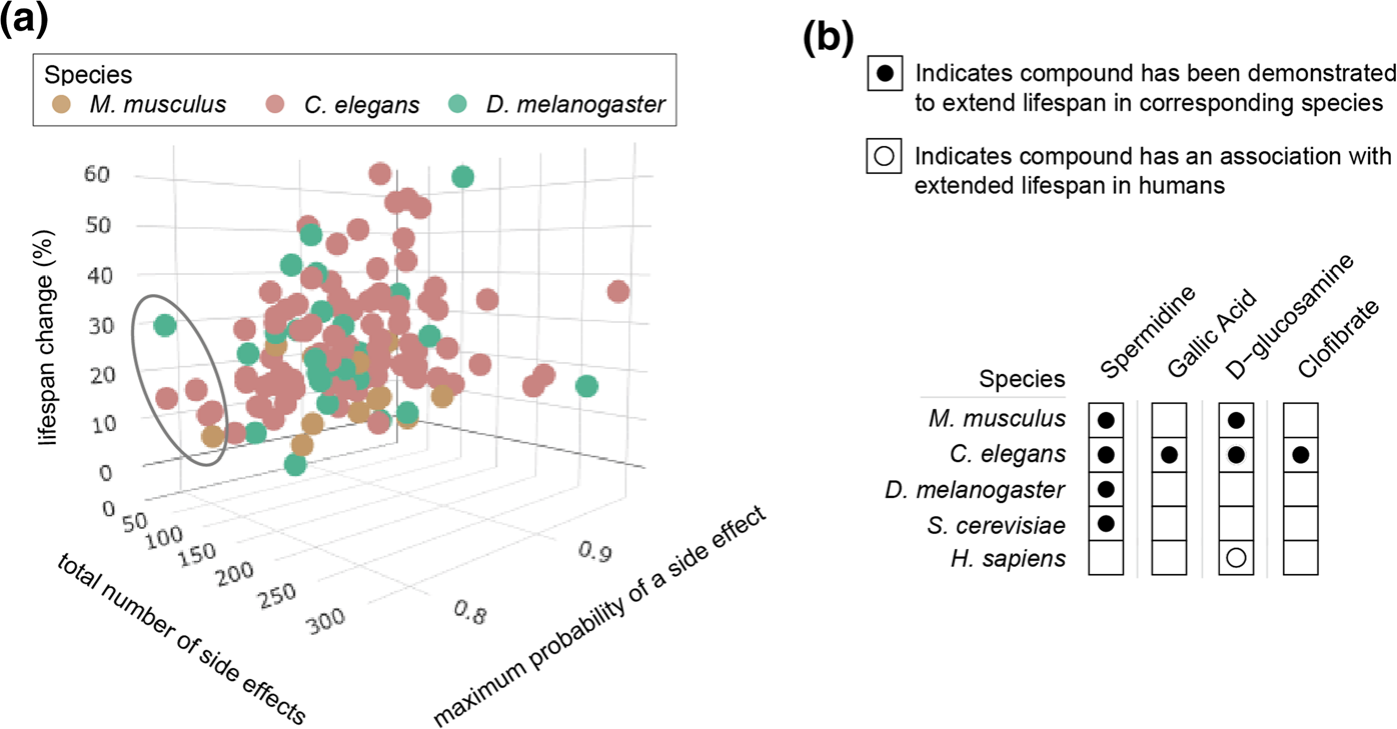
Identification of longevity compounds with minimized probabilities of side effects. **a** Three dimensional scatterplot of average lifespan change (%) a compound produces (y-axis) in model organisms (colored dots) compared to two summarizing parameters for a geroprotector’s side effects: the maximum probability of a side effect (x-axis), and the total number of side effects (z-axis). Shows a group of geroprotectors on the left-hand region of the plot, which are compounds with low side effect risks. The top compound within this group, with highest lifespan increase of these geroprotectors (x-axis), is spermidine (green point in encircled points). **b** The four compounds identified in (**a**), and the species in which longevity benefits have been confirmed. Compounds include the autophagy inducer spermidine (Eisenberg et al. 2009), the polyphenol gallic acid (Saul et al. 2011), the glycolysis inhibitor d-glucosamine (Weimer et al. 2014), and the lipid-lowering clofibrate (Brandstädt et al. 2013). The association that d-glucosamine has with extended lifespan in humans is additionally highlighted (see sect. “Discussion”)

Next, we asked what compounds based on these parameters may be most interesting to consider for clinical trial in humans. In order to assess which geroprotectors may be more relevant for human use, we plotted lifespan change for each compound, as compared to (1) the total side effects and (2) the maximum likelihood of a side effect for each compound, in a three-dimensional plot. We reasoned that this would reveal geroprotectors with lower chances of causing side effects, while maintaining a higher chance of benefit (Fig. 4a). Here a clear cluster emerged of four compounds that had both low total numbers of side effects and low maximum likelihoods of causing a side effect, while maintaining moderate lifespan extending potential. These geroprotectors included the autophagy inducer spermidine (Eisenberg et al. 2009), the polyphenol gallic acid (Saul et al. 2011), the glycolysis inhibitor d-glucosamine (Weimer et al. 2014), and the lipid-lowering PPAR agonist clofibrate (Brandstädt et al. 2013) (Fig. 4b).

Investigating these compounds further, we found spermidine’s highest likelihood of causing a side effect to be 0.78 for dizziness, out of 26 total side effects predicted. Both clofibrate (27 total side effects) and d-glucosamine (30 total side effects) had ‘rash’ as their top side effect (0.80 and 0.81 probability, respectively), while gallic acid (22 total side effects) had a 0.80 probability for ‘nausea’. None of these geroprotectors had a predicted risk of ‘death’. In order to put these observations in perspective, we compared these findings to two well-known geroprotectors, metformin and sirolimus (also known as rapamycin). Here, metformin had a highest likelihood of 0.94 to cause vomiting, out of 97 total side effects, while sirolimus had a highest likelihood of 0.98 for causing nausea, out of 253 total side effects. Notably, sirolimus included both ‘death’ and ‘cardiac failure’ as predicted side effects (probability of 0.74 and 0.81, respectively).

Of the four compounds we identified with minimized side effect risks, spermidine had the largest lifespan extension documented, increasing *D. melanogaster* lifespan by 30% (Eisenberg et al. 2009). All compounds had been tested in *C. elegans*, with an increased lifespan ranging from minimally 10% with gallic acid treatment (Saul et al. 2011), to maximally 16% with clofibrate treatment (Brandstädt et al. 2013) (Fig. 4b). To form a final short list of candidate geroprotectors, we selected the two amongst our list which had results reproduced in multiple species. These were spermidine and d-glucosamine, both of which also possessed lifespan data in mice, and d-glucosamine additionally possessed an association to longevity in humans (Fig. 4b) (Pocobelli et al. 2010; Bell et al. 2012; Kantor et al. 2018). Below we discuss these findings and recommend prioritization of these two compounds for further testing in model organisms and eventually controlled evaluation for human use as geroprotective compounds.

## Discussion

Many classes of compounds extend lifespan in model organisms (Carretero et al. 2015; Barardo et al. 2017; Partridge et al. 2020), including mTOR inhibitors (Johnson et al. 2013), HDAC inhibitors (Pasyukova and Vaiserman 2017; McIntyre et al. 2019), HSP90 inhibitors (Fuhrmann-Stroissnigg et al. 2017; Janssens et al. 2019), antibiotics (Houtkooper et al. 2013; Solis et al. 2018; Oxenkrug et al. 2012), and NAD+ boosters (Mouchiroud et al. 2013; Zhang et al. 2016). However, the transfer of these discoveries to benefit an aging human population has been slow. The most concrete case of the advancement of a geroprotector towards general human use has been established with the TAME clinical trial with metformin (Barzilai et al. 2016). The TAME trial (www.afar.org/research/TAME/) is a series of studies whereby 3000 individuals between the ages of 65 and 79 are followed in a 6-year period. The trials will test whether age-related chronic diseases, including heart disease, cancer, and dementia, can be reduced in metformin users as compared to placebo controls. The trial costs an estimated 75 million USD to conduct (De Grey 2019), and with such high inherent costs in testing longevity compounds, it is clear that an appropriate and strict triage of the many candidate geroprotectors available should take place. Additional compounds have been suggested by others for clinical use (Moskalev et al. 2016; Partridge et al. 2020), and indeed testing multiple compounds in humans would alleviate the inevitable failure rate that occurs from translating findings from model organisms to humans. Here we performed a systematic evaluation of the potential for the use of geroprotector’s in humans, and identified d-glucosamine and spermidine as top candidate compounds. Both are widely available to the public, and have gathered greater interest within the aging research community (Moskalev et al. 2016; Partridge et al. 2020).

d-Glucosamine (2-amino-2-deoxy-d-glucose, CAS 3416-24-8) inhibits glycolysis and is thought to act as a calorie restriction mimetic (Weimer et al. 2014). It notably differs from other calorie restriction mimetics such as 2-deoxy-d-glucose, as it extends lifespan in both worms and mice (Weimer et al. 2014), whereas the latter was found to extend lifespan in worms (Schulz et al. 2007) though shortened lifespan in rats (Minor et al. 2010). One study performed in yeast however did not find d-glucosamine to extend lifespan (Kaeberlein and Guarente 2002). In humans, the use of d-glucosamine has primarily been for the treatment of osteoarthritis (McAlindon et al. 2000; Reginster et al. 2001), and though mixed results for this have been observed (Wandel et al. 2010), it has nonetheless remained in use for decades due in part to its high tolerance and low side effects profile. While high-dose, short-term administration of d-glucosamine causes detrimental effects and a diabetes-like phenotype in humans (Monauni et al. 2000; Hawkins et al. 1996), chronic treatment with lower doses has produced no such effects, pointing towards a beneficial, blood glucose lowering result (Simon et al. 2011). Use of d-glucosamine in humans has been linked to lowered cardiovascular disease risk (Ma et al. 2019), and lowered mortality rates (Pocobelli et al. 2010; Bell et al. 2012; Kantor et al. 2018). Together this demonstrates that d-glucosamine possesses low side effect risks in humans, and is associated to human longevity (Pocobelli et al. 2010; Bell et al. 2012; Kantor et al. 2018) (Fig. 4b), and therefore should be prioritized for further investigation toward human geroprotection.

Spermidine (CAS 124-20-9) is a polyamine compound and increases lifespan in yeast, worms, flies, and mice, likely due to its autophagy boosting properties (Eisenberg et al. 2009; Eisenberg et al. 2016). In mice, it extended lifespan when treated both throughout life and only late in life (Eisenberg et al. 2016). Polyamines are synthesized in all animals, plants, and bacterial cells, and spermidine is one of the major polyamines in mammals, whose levels were found to decline with aging in humans (Scalabrino and Ferioli 1984). Recently, it has been suggested to relate to human longevity (Madeo et al. 2018), since individuals who reported higher dietary intake levels of spermidine were found to have longer life expectancies than those with lower levels (Kiechl et al. 2018). While dietary intake surveys may not necessarily prove the influence of a compound on lifespan in humans, together, these findings nonetheless suggest that spermidine should be prioritized for further investigation towards human geroprotection.

Our analysis revealed that compounds that induced a larger lifespan extension in worms concomitantly had higher risks of producing a side effect. This direct relationship between lifespan extension and side effect risk may be due to the fact that many geroprotectors act through a hormesis effect (Vaiserman 2014; Ristow 2014). Hormesis is the concept that certain toxic substances may possess a biphasic dose response in organisms. In this hormesis scenario, a high dose of the compound is detrimental, while a low dose causes the organism to elicit a protective stress response. The ensuing stress response is believed to neutralize or repair endogenously or environmentally produced damage in the organism which was already present, with the net result of this being an enhancement of survival (Calabrese et al. 2015). The property of a geroprotector to be toxic, acting through hormesis, intuitively would lead to greater probabilities of causing side effects. Our finding suggests that maximal lifespan extension may therefore not be the best readout when screening for novel geroprotectors in simple model organisms, since compounds identified in this manner may have limited translatability to humans. Furthermore, with respect to the development of hormetic geroprotectors it is worthwhile to note that combinations of two or more hormetic geroprotective compounds, especially when they act in or elicit the same molecular pathways, run the risk of no longer being beneficially hormetic, since in combination they may surpass the hormetic dosage and become toxic.

There are several limitations to consider in our study. Firstly, the compounds we considered were only those that overlapped between the databases of DrugAge for geroprotector entries and SEP-L1000 for predicted side effect listings, which constituted 124 out of the 417 possible drugs in DrugAge during the time of our analysis. Secondly, side effects were not differentiated for their severity; however, our final candidates were checked to be sure that terms such as ‘death’ or ‘cardiac arrest’ were not included. Thirdly, therapeutic dose is not estimated in our study, whereby side effects may not be relevant if the actual therapeutic dose proves to be lower for geroprotective effects. Nonetheless, these factors are all likely to produce false negatives rather than false positives when considering our short-list of candidate geroprotectors.

In light of potential false negatives resulting from our method, it may be interesting to contrast our results to a recently published overview of selected longevity compounds deemed worthwhile to test in humans (Partridge et al. 2020). This list included rapamycin, senolytics, metformin, acarbose, spermidine, NAD^+^ enhancers and lithium as a top tier of high potential geroprotectors. d-glucosamine, which was identified in our analysis, was present in a proposed secondary tier (Partridge et al. 2020). While rapamycin and metformin were described in our results above, our approach allows for the further evaluation of acarbose and lithium, both of which were included in our datasets. We found that both acarbose and lithium possessed 22 predicted side effects in total, whereby lithium possessed lower probabilities for each side effect, with the highest being an 0.86 probability for rash, followed by 0.78 for headache and 0.76 for nausea. Acarbose possessed higher probabilities for its side effects, starting at 0.97 for rash followed by 0.94 for both nausea and diarrhea. Based on our analyses, we suggest that lithium may also be an interesting addition for further evaluation, and indeed there is evidence that low dose lithium intake is associated to longevity in humans (Zarse et al. 2011; Fajardo et al. 2018).

A final point in regards to our work here is that each side effect is inherently dependent on the (epi)genetic background of the individual taking the drug. Differences between people produce different drug metabolism rates and off-target effects. This is likely attributable to unique profiles of genetic variation, exposomes, epigenomes, transcriptomes, proteomes, metabolomes, microbiomes, and interactomes and may produce greatly differing results between people for the same interventions (Ogino et al. 2013; Gao et al. 2018; Liao et al. 2010). These will all affect an individual’s aging trajectory, and the benefit or side effects they may have from a given geroprotector. Therefore, an era of ‘personalized geroprotection’ is likely to closely follow the testing of geroprotectors in humans.

To conclude, efforts such as the one presented in this work add to the body of literature that is currently being built to help prioritize geroprotectors for use in humans. Based on our analyses we specifically prioritize two compounds for their geroprotective effects and their transfer potential to human testing with minimized risks of side effects. While these compounds are widely available to the general public, it should be emphasized that this testing in humans should be performed in controlled trials in research-based settings.

## Data Availability

The data used in this study was derived from resources available in the public domain. The data that support the findings of this study are available at the DrugAge and SEP-L1000 databases, at https://genomics.senescence.info/drugs/ and http://maayanlab.net/SEP-L1000/, respectively.
